# Towards MRI-guided cardiac ablation procedures with no contrast agent: safety and efficacy considerations

**DOI:** 10.1186/1532-429X-18-S1-P213

**Published:** 2016-01-27

**Authors:** Michael Guttman, Aravindan Kolandaivelu, Sarah Fink, Henry Halperin, Daniel A Herzka

**Affiliations:** grid.21107.350000000121719311Johns Hopkins University, Baltimore, MD USA

## Background

Efforts are underway to perform MRI-guided cardiac ablations in the magnet. The chief motivations are: 1) the ability to see the effects of treatment, 2) improved catheter positioning during mapping and therapy, and 3) avoiding ionizing radiation and iodinated contrast agent from x-ray.

However, current efforts use gadolinium-based contrast agents (GBCAs) to obtain MR Angiogram (MRA) for anatomical context and late gadolinium enhancement (LGE) for enhancement of ablation lesions. There may be motivations to limit use of GBCAs, both in the specific context of an ablation procedure and in general use. The general use motivation is health-based, as the FDA is investigating the risk of gadolinium deposits in the brain after repeated use of GBCAs.

Another motivation is for accuracy in assessment of acute lesions, since wash-in/out kinetics make it difficult to infer lesion size from enhancement regions in LGE images, even with carefully chosen timing and imaging parameters. The typical LGE protocol requires waiting 10-15 minutes after contrast agent injection before T1w imaging. This protocol is both unpractical to run repeatedly and limited by contrast-agent dose.

It may be possible to perform this procedure without GBCAs. We focus on the acquisition of images for MRA, real-time navigation and lesion assessment. We present proof-of-concept data in swine left ventricle (LV).

## Methods

Healthy swine were placed in 1.5T MRI (Avanto, Siemens Medical Systems) and bright-blood whole-heart images were acquired using 3D navigator-gated TrueFISP, to serve as surrogate for MRA. Image window/level modification and clipping were used to suppress or remove background.

Ablations were performed using MR compatible catheters, navigating in the LV using real-time MRI. T1w images were acquired early after ablation.

3D TrueFISP was acquired with navigator-gating, voxel 0.6x0.6x3. For T1w: navigator-gating, voxel 0.7x0.7x2.5, flip=20, saturation prep, TR/TE/TI=498/1.4/440.

## Results

**Figure 1:**
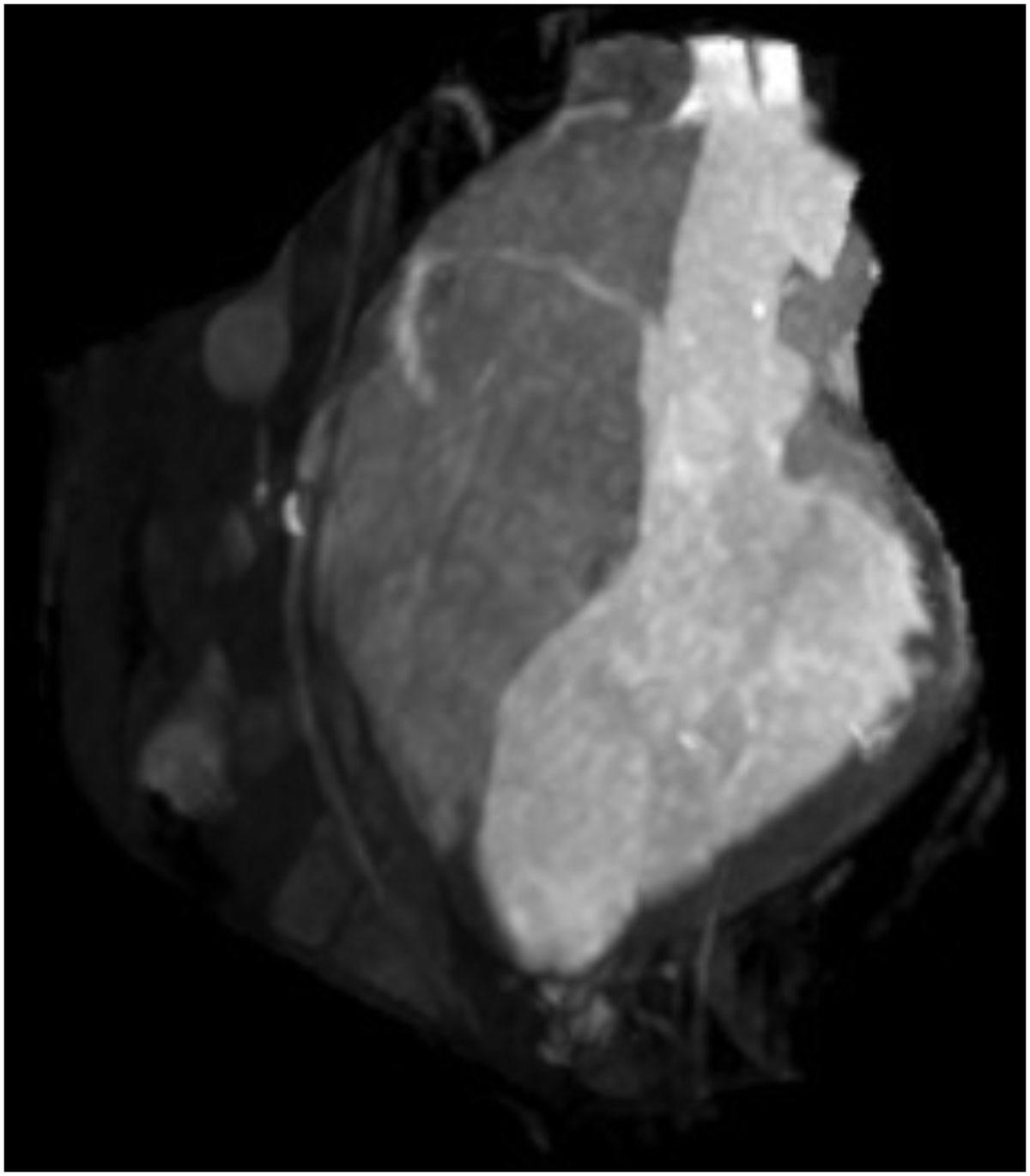
**3D TrueFISP image stack rendered as a MIP**.

A MIP rendering of an example bright-blood 3D TrueFISP image set is shown in Figure 1. The isolation of blood regions is inferior to that of typical contrast-enhanced MRA; however, it provided sufficient context while navigating in the LV when used together with real-time imaging.

**Figure 2:**
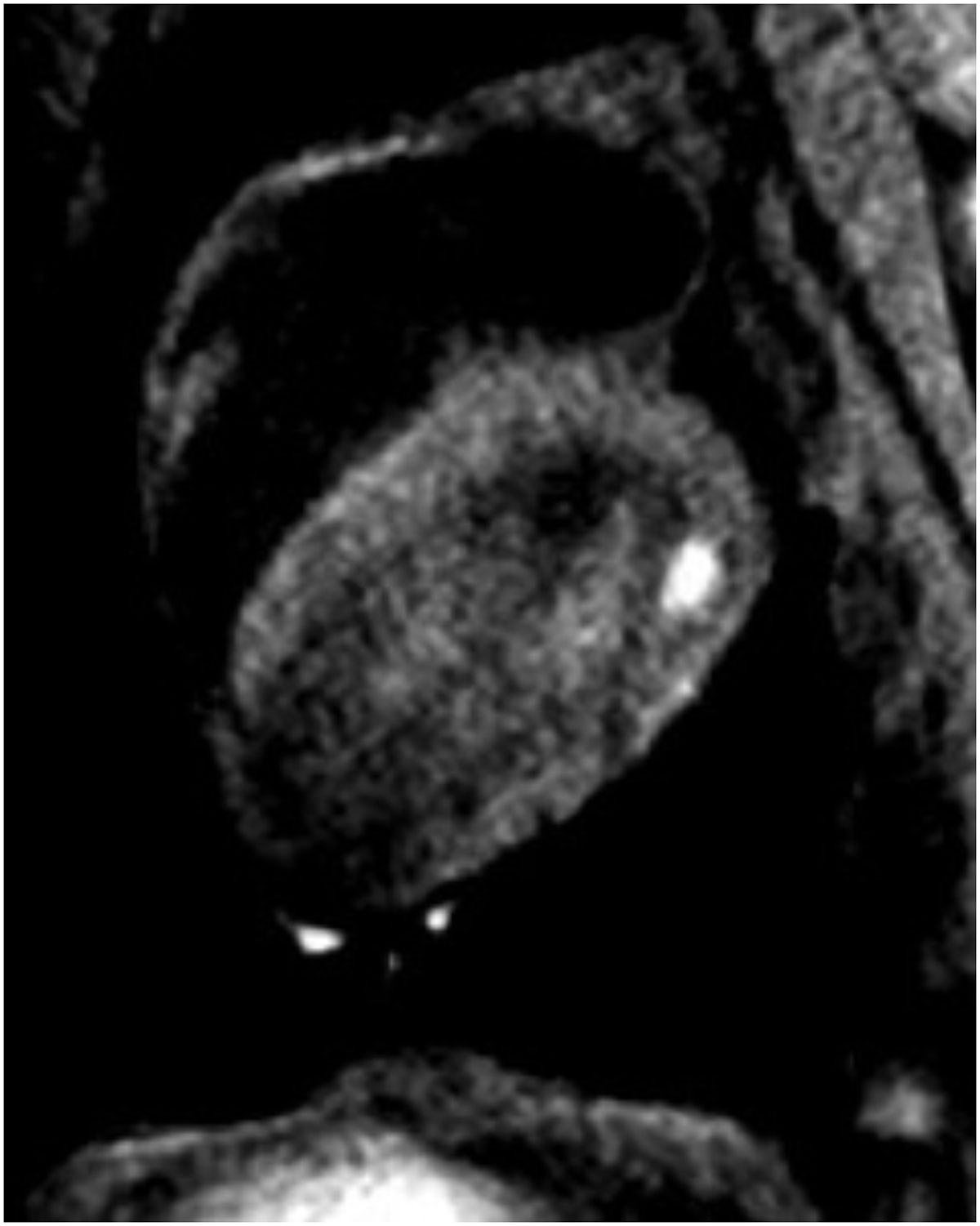
**T1w image acquired 5 minutes after ablation**.

Figure 2 shows a T1w image acquired 5 minutes after ablation. The lesion enhancement is readily seen in the LV wall.

## Conclusions

MRI-guided cardiac ablations in the LV may be possible without the use of any contrast agent. In the LV, 3D TrueFISP can be used as an MRA surrogate and lesions exhibit enhancement in T1w images soon after the ablation.

A non-contrast protocol may be beneficial, considering possible health effects of accumulated gadolinium and complications from wash-in/out kinetics on LGE image contrast. The non-contrast T1w images may be acquired many times during a procedure, and may assist in determining when a procedure is complete. This may have greater utility than an LGE scan, which can be performed only once or twice during a procedure.

